# Comorbidity of Temporomandibular Disorders, Anxiety, Depression, and Poor Sleep Quality in Episodic and Chronic Migraine: A Cross-Sectional Observational Study

**DOI:** 10.3390/jcm15155889

**Published:** 2026-07-28

**Authors:** Gema Torrijos-Gómez, Lidiane L. Florencio, María-Luz Cuadrado, Ignacio Ardizone, Jesús Porta-Etessam, Luana María Ramos Mendes

**Affiliations:** 1 School of Biomedical and Health Sciences, Universidad Alfonso X el Sabio, 28691 Villanueva de la Cañada, Madrid, Spain; gtorrgom@uax.es (G.T.-G.); jporta@yahoo.com (J.P.-E.); 2 Grupo de Investigación de Alto Rendimiento en Evaluación Multidimensional y Tratamiento del Dolor Crónico, Department of Physiotherapy, Ocupational Therapy, Rehabilitation and Physical Medicine, Universidad Rey Juan Carlos, 28922 Alcorcón, Spain; lidiane.florencio@urjc.es; 3 Department of Neurology, Hospital Clínico San Carlos, 28040 Madrid, Spain; 4 School of Medicine, Universidad Complutense de Madrid, 28040 Madrid, Spain; 5 School of Dentistry, Universidad Complutense de Madrid, 28040 Madrid, Spain; ignacioa@pdi.ucm.es; 6 Department of Neurology, Hospital Universitario Fundación Jiménez Díaz, 28040 Madrid, Spain; 7 Department of Health Sciences, Ribeirão Preto Medical School, University of São Paulo, São Paulo 14049-900, Brazil; luanamrmendes@usp.br

**Keywords:** migraine disorders, temporomandibular joint disorders, anxiety, depression, sleep quality

## Abstract

**Background/Objectives**: This study investigated the comorbidity between temporomandibular disorders (TMD) and migraine, distinguishing episodic migraine (EM) from chronic migraine (CM), and examined whether TMD presence in migraine patients is associated with more severe clinical or psychological features. **Methods**: A cross-sectional sample of 120 participants was evaluated, including 40 headache-free controls, 40 EM patients, and 40 CM patients. Demographic and clinical information was obtained through structured interviews. Participants completed the Headache Impact Test (HIT-6), State-Trait Anxiety Inventory (STAI), State-Trait Depression Questionnaire (ST-DEP), and Pittsburgh Sleep Quality Index (PSQI). Prevalence ratios (PR) analyzed the association between TMD and migraine and between TMD and migraine subtypes. Among migraine patients, headache frequency, medication use, headache impact, anxiety/depression symptoms, and sleep quality were compared between those with and without TMD. **Results**: TMD was significantly more common in migraine patients compared to controls (78.8% vs. 20.0%; PR = 3.94; 95% CI: 2.10–7.40). CM patients also showed a higher TMD prevalence than EM patients (95.0% vs. 62.5%; PR = 1.52; 95% CI: 1.18–1.95). Myalgia-related and mixed TMD were the most frequent subtypes. Migraine patients with TMD had increased headache frequency and higher acute medication intake compared to those without TMD. However, the presence of TMD was not associated with greater headache impact, anxiety, depression, or poorer sleep quality. **Conclusions**: Patients with migraine are almost four times more likely to present with TMD than individuals without headache, with an even stronger association observed in CM. Although TMD is associated with a greater headache burden, our findings suggest that it may not be accompanied by worse psychological or sleep outcomes.

## 1. Introduction

Headache disorders appear among the most burdensome of all health conditions, affecting nearly 2.9 billion individuals globally according to the Global Burden of Disease Study 2023 [1]. Migraine makes a major contribution, accounting for the highest number of disability-adjusted life years (DALYs) among neurological disorders [2]. Understanding migraine phenotypes and the factors associated with more severe clinical presentations is an important challenge in the field [3]. One approach to addressing this challenge is to further explore the relationship between migraine and its comorbid conditions.

Recent systematic reviews have confirmed the comorbidity between migraine and temporomandibular disorders (TMD) [4,5,6]. Migraine is the most common primary headache associated with TMD [5]. Several studies have shown that patients with migraine are more likely to present with TMD than control subjects [4,5,6], and this association is particularly strong in cases with chronic migraine (CM). However, most studies do not differentiate between migraine subtypes when assessing this comorbidity [4].

Migraine and TMD are chronic pain conditions that share other comorbidities such as anxiety, depression, and sleep disorders [7,8,9,10,11,12,13]. The interaction between these two overlapping painful conditions has the potential to promote a greater impact, worse clinical presentation and to challenge their management [14].

In patients with TMD, the reported findings are conflicting. The presence of migraine has been associated with higher levels of TMD or chronic pain-related disability, greater pain intensity, lower pressure pain thresholds, and increased psychological distress [15,16,17]. Migraine may also act as a confounding factor contributing to the variability in Central Sensitization Inventory scores in patients with TMD [18]. However, the presence of migraine does not appear to exacerbate psychological disorders, such as depression or anxiety, among patients with TMD [17,19].

In patients with migraine, the presence of TMD was associated with greater cutaneous allodynia and sensitization, as well as a worse impact of headaches [15,20,21]. On the other hand, women with chronic, but not episodic, migraine exhibited a higher risk of more severe manifestations of TMD than non-migraine controls [22]. However, to the best of our knowledge, information on psychosocial factors and TMD comorbidity in patients with migraine remains scarce.

The aim of this study was to analyze the prevalence of TMD in migraine patients attending a specialized headache center, considering migraine subtypes (episodic or chronic). Moreover, the study sought to determine whether the presence of TMD in migraine patients is associated with more severe clinical manifestations and adverse psychological factors. We hypothesized that migraine would be associated with a higher prevalence of TMD and that this comorbidity would be associated with more severe clinical manifestations, greater anxiety and depression, and poorer sleep quality.

## 2. Materials and Methods

This is a cross-sectional observational study, which followed the recommendations of the Strengthening the Reporting of Observational Studies in Epidemiology (STROBE) Statement [23]. The study protocol was approved by the Research Ethics Committee of Hospital Clínico San Carlos, Madrid, Spain (code 15/434-E).

Patients were consecutively recruited from the Headache Unit of Hospital Clínico San Carlos from February 2017 to January 2018. Inclusion criteria were: (1) age 18–65 years old; (2) both sexes; (3) migraine diagnosis according to the beta version of the third International Classification of Headache Disorders (ICHD-3-beta) [24], and (4) absence of other headache diagnosis (except for headache secondary to TMD or medication overuse headache). The beta version of the ICHD-3 was the most up-to-date diagnostic classification available at the time. The main migraine-related change introduced in the final ICHD-3 concerned the diagnostic criteria for migraine with aura, which showed greater specificity than those in the beta version [25,26]. However, the core definition of chronic migraine (CM), on which the subgroup analysis was based, remained essentially unchanged [24,25]. For the control group, we recruited participants paired by sex and by age (±2 years) who did not present migraine or other headaches (except for headache secondary to TMD). Exclusion criteria were: (1) pregnancy; (2) alcohol abuse or illicit drug use; (3) history of serious psychiatric illnesses (psychotic episodes, major depression); (4) history of surgery or trauma to the cranial or cervical region; (5) treatment with botulinum toxin in the last three months; (6) treatment with physical therapy in the masticatory muscles; (7) serious systemic diseases; (8) any orthodontic or dental treatment active at the time of the study, and (9) inability to understand and complete the informed consent or psychological tests, or to keep an adequate headache diary. All participants included in the study gave their written informed consent.

Patients were first assessed by a neurologist to confirm migraine diagnosis and exclude other types of headaches. According to the ICHD-3-beta [24], a diagnosis of chronic migraine (CM) was made if patients experienced headaches on more than 15 days per month for at least three months, with migraine features on at least eight days per month. If headache frequency was lower, patients were diagnosed with episodic migraine (EM). They then completed a structured interview covering demographic and clinical data, including age, sex, body mass index, years with migraine, number of days with headache in the last month, number of days with moderate or severe headache in the last month, and acute treatment days in the last month.

Patients with migraine also completed the Headache Impact Test (HIT-6) [27,28], while all participants, including patients and controls, completed the State-Trait Anxiety Inventory (STAI) [29], the State-Trait Depression Questionnaire (ST-DEP) [30], and the Pittsburgh Sleep Quality Index (PSQI) [31,32]. All these questionnaires were self-administered. Finally, all participants were assessed by a dentist with expertise in TMD who administered the axis I of the Diagnostic Criteria for Temporomandibular Disorders (DC/TMD) [33].

### 2.1. Questionnaires

The HIT-6 is a 6 item-questionnaire that verifies headache-related impact. The total score varies between 36 and 78 points. Headache-related impact is classified as little or no impact (≤49 points), some impact (50–55 points), considerable impact (56–59 points), and severe impact (≥60 points) [27,28].

The STAI is a tool that assesses anxiety in two dimensions: state anxiety (STAI-S), which refers to a temporary state of anxiety that can fluctuate over time, and trait anxiety (STAI-T), which indicates a relatively stable anxious tendency that characterizes individuals who tend to perceive situations as threatening. Each scale has 20 items [29]. Anxiety level of the STAI-S was classified by sex as: mild (men = 0–8 points; women = 0–10 points), low to moderate (men = 9–15 points; women = 11–16 points), moderate to high (men = 16–22 points; women = 17–26 points), or high (men = 23–60 points; women = 27–60 points). Anxiety level of the STAI-T was classified as: mild (men = 0–11 points; women = 0–16 points), low to moderate (men = 12–18 points; women = 17–23 points), moderate to high (men = 19–25 points; women = 24–30 points), or high (men = 26–60 points; women = 31–60 points).

The ST-DEP is a psychological instrument designed to measure the affective component of depression, by assessing both its current intensity (state) and long-term prevalence (trait). The questionnaire has two scales of 10 items each, Trait and State, with two subscales each: dysthymia (negative affectivity) and euthymia (positive affectivity). The final score can range from 10 to 40 [30]. For men, state depression was classified as mild (10–13 points), low to moderate (14–16 points), moderate to high (17–20 points), or high (21–40 points); trait depression was classified as mild (10–13 points), low to moderate (14–17 points), moderate to high (18–21 points), or high (22–40 points). For women, state depression was classified as mild (10–13 points), low to moderate (14–17 points), moderate to high (18–22 points), or high (23–40 points); trait depression was classified as mild (10–14 points), low to moderate (15–19 points), moderate to high (20–24 points), or high (25–40 points).

The PSQI is a self-report questionnaire that assesses sleep quality over the past month. The scale includes seven components: subjective sleep quality, sleep latency, sleep duration, habitual sleep efficiency, sleep disturbances, use of sleep medication, and daytime dysfunction. Each component is rated from 0 to 3. The sum of these components yields a global score ranging from 0 to 21, where a total score greater than 5 indicates poor sleep quality, providing a sensitive and specific measure relative to clinical and laboratory evaluations [31,32].

### 2.2. Evaluation of Temporomandibular Disorders

The Axis I of the DC/TMD includes a 6-item TMD Pain Screener, the DC/TMD Symptom Questionnaire, and a standardized physical examination. The examination involves initial observation, joint and muscle palpation, evaluation of mandibular movement and function, assessment of pain with palpation and mandibular movement, and verification of joint noises and/or locking [33]. The Axis I diagnostic algorithm classifies the most common TMD into two major groups: pain-related TMDs (local myalgia, myofascial pain, myofascial pain with referral, arthralgia, and headache attributed to TMD) and intra-articular TMDs (disc displacement disorders, degenerative joint disease, and subluxation) [33]. The DC/TMD presents adequate criterion validity for the most common pain-related disorders (sensitivity ≥ 0.86, specificity ≥ 0.98) [33]. In this study, we investigated the presence of pain-related TMDs, categorizing them as: (1) myalgia-related, including local myalgia (with pain limited to the area of palpation), myofascial pain (with pain spreading beyond the area of palpation but not beyond the muscle’s boundaries), and myofascial pain with referral (with pain felt in distant structures); (2) arthralgia; (3) mixed (myalgia and arthralgia), and (4) headache attributed to TMD.

### 2.3. Statistical Analysis

Statistical analyses were performed using IBM SPSS Statistics, version 31.0 (IBM Corp., Armonk, NY, USA). Descriptive data are presented as frequencies and percentages for categorical variables, and as means and standard deviations (SD) for continuous variables. Differences in categorical variables between patients and controls were assessed using the chi-squared test. Except for 2 × 2 comparisons, post hoc analyses were performed using adjusted standardized residuals when the initial chi-square test was statistically significant to understand which specific cells or categories contributed most to that significance. Student’s *t*-test for independent samples was used to compare cases and controls for continuous variables, while one-way analysis of variance (ANOVA) was applied to compare the CM, EM, and control groups. Additionally, the prevalence ratio (PR) and its 95% confidence interval (95% CI) were calculated to determine the strength of the association between TMD and migraine, and between TMD and the two migraine subtypes (CM and EM). Among patients with migraine, the chi-square test or Student’s *t*-test was used to compare clinical presentation, headache-related impact, symptoms of anxiety and depression, and sleep quality between those with and without TMD, according to the variable. Because the sample sizes of the subgroups defined by TMD status were fixed rather than determined a priori, a post hoc sensitivity analysis was performed using G*Power 3.1.9.6 (Heinrich-Heine Universität, Düsseldorf, Germany). Minimum detectable effect sizes were estimated assuming α = 0.05 and 80% power and were expressed as Cramér’s V for χ^2^ tests and Cohen’s d for independent-samples t-tests. As an additional analysis, we fitted separate logistic regression models to assess the association between the HIT-6, STAI-S, STAI-T, S-DEP, T-DEP, and PSQI classifications and TMD status (absence versus presence), adjusting for headache frequency (headache days per month). A level of significance of 0.05 was adopted in all statistical tests.

## 3. Results

**Figure 1:**
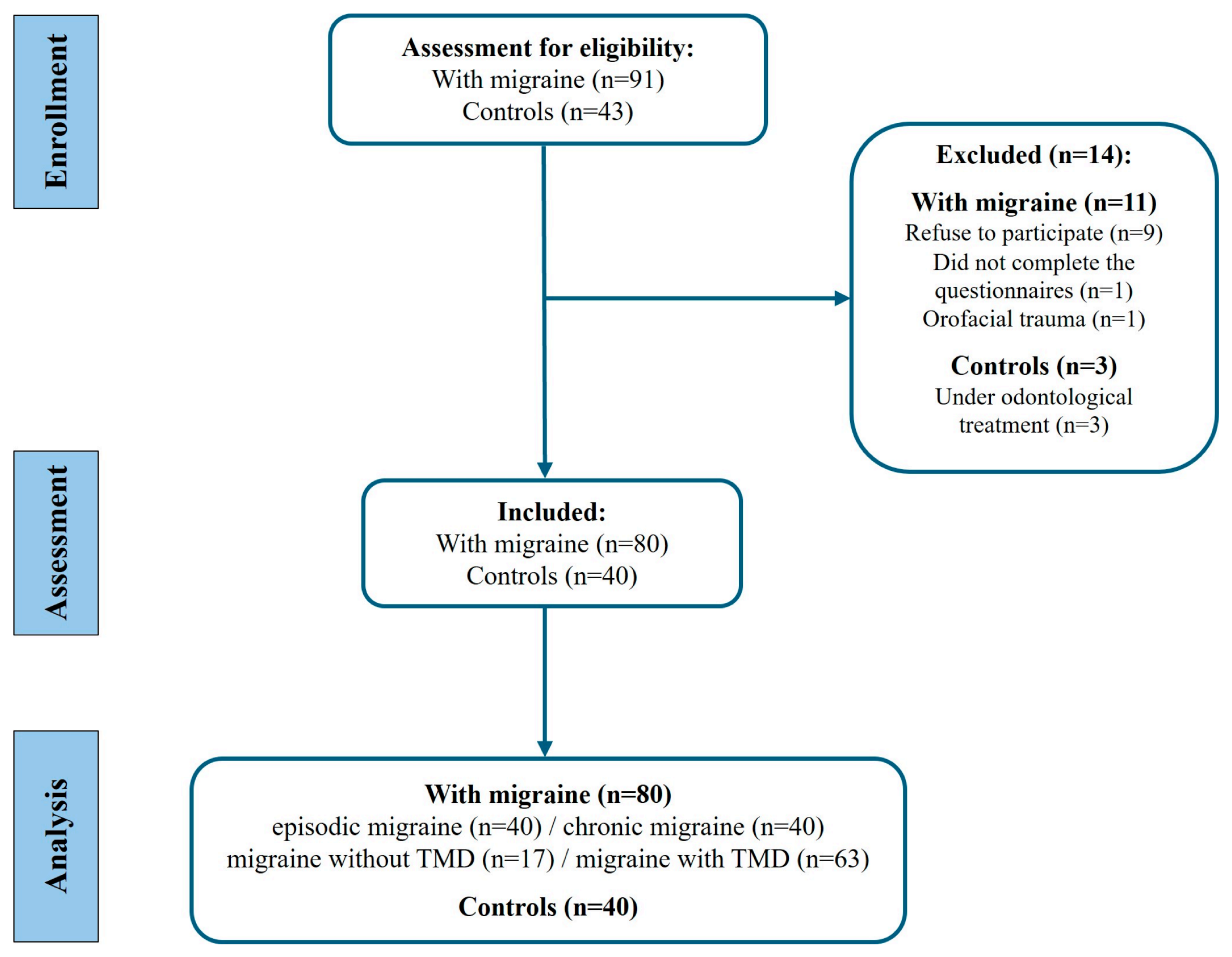
Flow diagram of the study (TMD: temporomandibular disorders).

A total of 120 participants were assessed, 80 with migraine and 40 controls (Figure 1). Table 1 presents the descriptive data of the total sample (*n* = 120), stratified as migraine group (*n* = 80) and control group (*n* = 40). In the migraine group, there were 13 men and 67 women with a mean age of 39.9 (SD 11.5) years old, while in the control group there were 7 men and 33 women with a mean age of 38.0 (SD 11.2) years old. The presence of migraine was associated with anxiety state (*X*^2^(3) = 8.71; *p* = 0.03), depression state (*X*^2^(3) = 10.39; *p* = 0.02), and poor sleep quality (*X*^2^(1) = 5.93; *p* = 0.03).

Descriptive data stratified as CM (*n* = 40), EM (*n* = 40) and controls (*n* = 40) are presented in Table 2. As expected by the diagnosis, there were differences between EM and CM on headache frequency, headache-related impact, and acute medication intake, but not for years with migraine. Significant differences were observed in sleep quality. For the global PSQI score, differences between groups were significant (F = 10.418; *p* < 0.001), with the CM group showing poorer sleep quality compared with the control group (mean difference: 3.95; 95% CI: 1.84–6.06; *p* < 0.001). In addition, the distribution of participants with good or poor sleep quality differed significantly between groups (χ^2^(2) = 6.81; *p* = 0.03). Post hoc analysis with adjusted standardized residuals revealed that the CM group had a significantly higher than expected frequency of poor sleep quality (z = 2.0; *p* < 0.01), whereas the control group showed a significantly higher than expected frequency of good sleep quality (z = 2.4; *p* < 0.001). After Bonferroni correction, these differences remained significant.

Regarding TMD prevalence, a higher prevalence was observed in the migraine group compared with controls without headache (78.8% vs. 20.0%; PR: 3.94; 95% CI: 2.10–7.40). When migraine subgroups were analyzed separately, both patients with EM (PR: 3.12; 95% CI: 1.61–6.07) and those with CM (PR: 4.75; 95% CI: 2.55–8.86) showed a higher prevalence of TMD than the control group. Furthermore, patients with CM presented a higher prevalence of TMD when compared with those with EM (95.0% vs. 62.5%; PR: 1.52; 95% CI: 1.18–1.95). The most frequent TMD subtypes were myalgia-related and mixed TMD (Table 3).

The migraine with TMD group experienced more headache days per month, more moderate-to-severe headache days per month, and higher acute medication intake per month than the migraine without TMD group (*p* < 0.05; Table 4). However, there were no significant differences in headache-related impact, depression, anxiety or sleep quality when the migraine group was stratified in function of the presence of TMD (*p* > 0.05; Table 4). These findings remained unchanged after adjustment for headache frequency in the regression analyses (See Supplementary Files).

## 4. Discussion

In our study, a comorbidity between migraine and TMD patients was confirmed, with greater association observed for the CM group when compared to controls or those with EM. Patients with migraine and TMD had greater headache frequency and acute medication intake than those with migraine without TMD. However, TMD comorbidity in our patients with migraine was not significantly associated with poorer psychological or sleep outcomes.

The association between migraine and TMD has been justified by the shared anatomical and physiological relationship mediated by the trigeminal nociceptive system [14]. Although it seems to be a well-explored relationship, few studies have assessed this comorbidity differentiating EM from CM. In a recent meta-analysis comparing the risk of TMD in migraine and tension-type headache patients versus non-headache controls, Bizzari et al. [4] found that TMD prevalence in EM was evaluated in only one study [22], while the CM subgroup included just two studies [22,34]. Both cited studies reported data from a specialized headache center from Brazil. Florencio et al. [22] reported higher TMD prevalence rates than those observed in our sample for both the EM (78% vs. 63%), and CM (100% vs. 95%) groups, as well as among controls (54% vs. 20%). These differences may be partly because the study only included women, since TMD are more prevalent in women than in men [35]. Otherwise, the PR and its confidence interval of 3.94 (95% CI: 2.10–7.40) observed for our migraine group is comparable to the odds ratio (OR) of 4.01 (95% CI: 2.61–6.18) obtained in the pooled analysis by Bizzari et al. [4] and of 6.08 (95% CI: 4.80–7.68) observed by Dias et al. [6], supporting the external validity of our data. The data reported in the systematic review by Dibello et al. [5] could not be directly compared with our results, as their analyses were presented according to TMD subtypes, which were not differentiated in our sample.

As migraine and TMD are overlapping pain conditions, their co-occurrence would be expected to result in a greater burden and worse clinical presentations [36,37]. However, this was not fully supported by our results. The negative interaction between two comorbid conditions may not be a general rule for all painful disorders. TMD presentation appears to be affected when it co-occurs with migraine, particularly in terms of pain intensity, disability, and pain thresholds [15,16,17], but not in terms of psychological features [17,19]. In patients with migraine, the comorbidity with TMD impacts on sensitization as demonstrated for cutaneous allodynia, pressure pain threshold, and heat/cold pain thresholds [15,20], but to the best of our knowledge, there is no further information regarding other clinical variables.

Regarding anxiety and depression, Viñals-Narváez et al. [19] reported that migraine and TMD patients may present common aspects but distinct profiles. Situational anxiety together with a lack of coping strategies could be more related to TMD myalgia, while the anxiety trait together with depression might be more related to migraine. Interestingly, these authors assessed migraine and TMD in isolation or combined and did not find any profile related to the combined conditions.

An important aspect that should be considered is that most of the studies that assess the impact of migraine on TMD characteristics do not account for the frequency of migraine [15,16,17,19]. In fact, CM can be a confounder when assessing this comorbidity. In our study, the characteristics that differed between migraine patients with and without TMD were headache frequency and acute medication intake, and both are directly associated with CM. The PSQI differences between migraine and controls seems also to be influenced by the CM group. These findings reinforce the need to distinguish CM from EM when examining the association with other conditions and the impact of their comorbidity with migraine.

On the other hand, Mengi and Uygunoglu [21] reported greater cutaneous allodynia and a higher headache-related impact in patients with CM who also had TMD and were not receiving preventive treatment. These findings suggest that TMD may confer an additional burden in certain migraine subgroups, which warrants further investigation in future studies. In the present study, we were unable to analyze EM and CM separately in relation to TMD due to the small sample size, particularly for CM without TMD (*n* = 2). This imbalance limits the internal validity of subgroup comparisons. Therefore, the association between migraine-related clinical severity and TMD comorbidity should be interpreted cautiously and confirmed in larger, adequately powered studies including sufficient numbers of patients with EM and CM, both with and without TMD.

Another area for future research, beyond the variables evaluated in this study, is the potential impact of medication overuse on this clinical interaction [25]. In addition, future studies may explore the contribution of craniofacial and occlusal characteristics, including factors such as dental crowding, within broader multifactorial models of TMD–migraine comorbidity. Such investigations may help clarify whether these factors influence symptom presentation, although current evidence does not support a primary role for them in TMD or in oral health-related quality of life [38,39,40].

The limitations of the present study should be acknowledged. First, the sample characteristics should be considered when generalizing the findings. The sample was recruited from a specialized headache center, which may include patients with more severe clinical presentation than samples obtained from the general population. Also, most of the sample were women. Although this reflects the distribution expected for migraine and despite sex balance across the study groups, we cannot guarantee that similar results would be observed in a subgroup composed only of men. In addition, the sample size may have limited statistical power to detect smaller effects or subgroup-specific differences. The post hoc sensitivity analysis of comparisons based on the presence or absence of TMD showed that the available sample size only provided sufficient power to detect large between-group effects with Student’s t-tests and moderate-to-large effects with χ^2^ tests. Therefore, some non-significant findings should be interpreted with caution, as they may reflect insufficient power rather than the absence of a true association. Future studies with larger and more balanced TMD and non-TMD subgroups are warranted. Finally, the cross-sectional study design only allows us to observe associations and describe their magnitude in terms of prevalence ratios. Causal relationships cannot be inferred, and the real impact of one new condition on the clinical course of the other can only be clarified through longitudinal studies. Conversely, the strengths of this study were the use of gold standards to define cases, ICHD for migraine and DC/TMD for TMD.

## 5. Conclusions

In our sample, patients with migraine were nearly four times more likely to have TMD than individuals without headache, and this likelihood was significantly higher among patients with CM than among those with EM. However, within the migraine group, the presence of TMD was not significantly associated with headache-related impact, anxiety, depression, or poor sleep quality.

## Figures and Tables

**Table 1:** Sample characteristics of patients with migraine and controls without headache.

	Control (*n* = 40)	Migraine (*n* = 80)	*p* Value ^a^
Age (years)	38.0 (11.2)	39.9 (11.5)	0.39
Sex			
Men	7 (17.5%)	13 (16.2%)	0.86
Women	33 (82.5%)	67 (83.8%)	
Body mass index	23.9 (3.9)	24.1 (3.9)	0.81
State anxiety (STAI-S)			
Mild	14 (35.0%)	26 (32.5%)	**0.03**
Low-moderate	13 (32.5%)	15 (18.8%)	
Moderate-high	10 (25.0%)	15 (18.8%)	
High	3 (7.5%)	24 (30.0%)	
Trait anxiety (STAI-T)			
Mild	17 (42.5%)	24 (30.0%)	0.39
Low-moderate	10 (25.0%)	22 (27.5%)	
Moderate-high	8 (20.0%)	15 (18.8%)	
High	5 (12.5%)	19 (23.8%)	
State depression (S-DEP)			
Mild	10 (25.0%)	18 (22.5%)	**0.02**
Low-moderate	20 (50.0%)	22 (27.5%)	
Moderate-high	7 (17.5%)	15 (18.8%)	
High	3 (7.5%)	25 (31.2%)	
Trait depression (T-DEP)			
Mild	20 (50.0%)	30 (37.6%)	0.08
Low-moderate	13 (32.5%)	17 (21.2%)	
Moderate-high	4 (10.0%)	17 (21.2%)	
High	3 (7.5%)	16 (20.0%)	
PSQI score	6.3 (3.0)	9.06 (4.4)	**<0.001**
PSQI classification			
Good sleep quality	20 (50.0%)	22 (27.5%)	**0.03**
Poor sleep quality	20 (50.0%)	58 (72.5%)	

Data are presented as mean (standard deviation) or as frequency (percentage). ^a^ Quantitative variables were compared using Student’s *t*-test for independent samples (control group vs. migraine group); qualitative variables were contrasted using X^2^ test. Bold *p*-values indicate statistically significant differences (*p* < 0.05). STAI-S: State–Trait Anxiety Inventory—state subscale; STAI-T: State–Trait Anxiety Inventory—trait subscale; S-DEP: State-Trait Depression Questionnaire—state subscale; T-DEP: State-Trait Depression Questionnaire—trait subscale; PSQI: Pittsburgh Sleep Quality Index.

**Table 2:** Sample characteristics and clinical data of patients with episodic migraine, chronic migraine and controls without headache.

	Control (*n* = 40)	EM (*n* = 40)	CM (*n* = 40)	*p* Value ^a^
Age (years)	38.0 (11.2)	37.0 (11.2)	42.9 (11.2)	**0.05**
Sex				
Men	7 (17.5%)	7 (17.5%)	6 (15.0%)	0.94
Women	33 (82.5%)	33 (82.5%)	34 (85.5%)	
Body mass index	24.0 (3.9)	23.7 (3.3)	24.6 (4.4)	0.58
Years with migraine		17.0 (12.1)	20.2 (11.0)	0.11
Headache days/month		4.6 (2.9)	21.4 (5.8)	**<0.001**
Moderate-severe headache days/month		2.2 (1.8)	13.4 (5.4)	**<0.001**
Acute medication days/month		4.0 (3.0)	15.3 (6.8)	**<0.001**
HIT-6				
No impact		1 (2.5%)	0	**0.03**
Some impact		2 (5%)	3 (7.5%)	
Considerable impact		15 (37.5%)	5 (12.5%)	
Severe impact		22 (55.0%)	32 (80.0%)	
State anxiety (STAI-S)				
Mild	14 (35.0%)	15 (37.5%)	11 (27.5%)	0.12
Low-moderate	13 (32.5%)	6 (15.0%)	9 (22.5%)	
Moderate-high	10 (25.0%)	8 (20.0%)	7 (17.5%)	
High	3 (7.5%)	11 (27.5%)	13 (32.5%)	
Trait anxiety (STAI-T)				
Mild	17 (42.5%)	16 (40.0%)	8 (20.0%)	0.35
Low-moderate	10 (25.0%)	9 (22.5%)	13 (32.5%)	
Moderate-high	8 (20.0%)	7 (17.5%)	8 (20.0%)	
High	5 (12.5%)	8 (20.0%)	11 (27.5%)	
State depression (S-DEP)				
Mild	10 (25.0%)	10 (25.0%)	8 (20.0%)	0.06
Low-moderate	20 (50.0%)	13 (32.5%)	9 (22.5%)	
Moderate-high	7 (17.5%)	6 (15.0%)	9 (22.5%)	
High	3 (7.5%)	11 (27.5%)	14 (35.0%)	
Trait depression (T-DEP)				
Mild	20 (50.0%)	16 (40.0%)	14 (35.0%)	0.08
Low-moderate	13 (32.5%)	11 (27.5%)	6 (15.0%)	
Moderate-high	4 (10.0%)	8 (20.0%)	9 (22.5%)	
High	3 (7.5%)	5 (12.5%)	11 (27.5%)	
PSQI score	6.3 (3.0)	7.9 (4.0)	10.2 (4.5)	**<0.001**
PSQI classification				
Good sleep quality	20 (50.0%)	13 (32.5%)	9 (22.5%)	**0.03**
Poor sleep quality	20 (50.0%)	27 (67.5%)	31 (77.5%)	

Data are presented as mean (standard deviation) or frequency (percentage). ^a^ Quantitative variables were compared using one-way ANOVA (CM vs. EM vs. controls) or Student’s *t*-test for independent samples (CM vs. EM); qualitative variables were contrasted using X^2^. Bold *p*-values indicate statistically significant differences (*p* < 0.05). EM: episodic migraine; CM: chronic migraine; HIT-6: Headache Impact Test; STAI-S: State–Trait Anxiety Inventory—state subscale; STAI-T: State–Trait Anxiety Inventory—trait subscale; S-DEP: State-Trait Depression Questionnaire—state subscale; T-DEP: State-Trait Depression Questionnaire—trait subscale; PSQI: Pittsburgh Sleep Quality Index.

**Table 3:** Prevalence and prevalence ratio of temporomandibular disorders in the different study groups.

	Control (*n* = 40)	Migraine, All (*n* = 80)	EM (*n* = 40)	CM (*n* = 40)
TMD prevalence	8 (20.0%) ^a,b^	63 (78.8%) ^a^	25 (62.5%) ^b^	38 (95.0%) ^b^
PR (95%CI)				
Controls as reference		3.94 (2.10–7.40)	3.12 (1.61–6.07)	4.75 (2.55–8.86)
EM as reference				1.52 (1.18–1.95)
TMD subtypes				
Myalgia-related	5 (12.5%)	34 (42.5%)	17 (42.5%)	17 (42.5%)
Arthralgia	0	1 (1.3%)	1 (2.5%)	0
Mixed ^c^	2 (5.0%)	23 (28.8%)	6 (15.0%)	17 (42.5%)
Headache attributed to TMD	1 (2.5%)	5 (6.3%)	1 (2.5%)	4 (10.0%)

Data are presented as frequency (percentage). ^a^ *p* value from X^2^ comparing control vs. migraine: <0.001; ^b^ *p* value from X^2^ comparing control vs. EM vs. CM: <0.001; ^c^ Mixed TMD was considered when participants presented both myalgia-related TMD and arthralgia. TMD: temporomandibular disorders; PR: prevalence ratio; EM: episodic migraine; CM: chronic migraine.

**Table 4:** Sample characteristics and clinical data of patients with migraine stratified by the presence of temporomandibular disorders.

	Migraine Without TMD (*n* = 17)	Migraine with TMD (*n* = 63)	*p* Value ^a^	Post-Hoc Sensitivity Analysis ^b^
Age (years)	35.8 (11.3)	41.0 (11.4)	0.05	*d* = 0.78
Sex			0.46	*V* = 0.31
Men	4 (23.5%)	9 (14.3%)		
Women	13 (76.5%)	54 (85.7%)		
Body mass index	22.6 (2.8)	24.5 (4.1)	**0.04**	*d* = 0.78
Years with migraine	17.9 (13.8)	18.8 (11.1)	0.39	*d* = 0.78
Headache days/month	6.8 (5.2)	14.7 (9.8)	**0.001**	*d* = 0.78
Moderate-severe headache days/month	4.1 (4.7)	8.8 (7.1)	**0.006**	*d* = 0.78
Acute medication days/month	5.4 (5.4)	10.8 (7.9)	**0.005**	*d* = 0.78
HIT-6				
Little or no impact	0	1 (1.6%)	0.25	*V* = 0.37
Some impact	0	5 (7.9%)		
Considerable impact	7 (41.2%)	13 (20.6%)		
Severe impact	10 (58.8%)	44 (69.8%)		
State anxiety (STAI-S)				
Mild	7 (41.2%)	19 (30.2%)	0.83	*V* = 0.37
Low-moderate	2 (11.8%)	13 (20.6%)		
Moderate-high	3 (17.6%)	12 (19.0%)		
High	5 (29.4%)	19 (30.2%)		
Trait anxiety (STAI-T)				
Mild	9 (52.9%)	15 (23.8%)	0.15	*V* = 0.37
Low-moderate	3 (17.6%)	19 (30.2%)		
Moderate-high	3 (17.6%)	12 (19.0%)		
High	2 (11.9%)	17 (27.0%)		
State depression (S-DEP)				
Mild	5 (29.4%)	13 (20.6%)	0.63	*V* = 0.37
Low-moderate	6 (35.3%)	16 (25.4%)		
Moderate-high	2 (11.8%)	13 (20.6%)		
High	4 (23.5%)	21 (33.4%)		
Trait depression (T-DEP)				
Mild	7 (41.2%)	23 (36.5%)	0.08	*V* = 0.37
Low-moderate	5 (29.4%)	12 (19.0%)		
Moderate-high	5 (29.4%)	12 (19.0%)		
High	0 (0.0%)	16 (25.4%)		
PSQI score	7.6 (3.5)	9.5 (4.6)	0.06	*d* = 0.78
PSQI classification				
Good sleep quality	5 (29.4%)	17 (27.0%)	0.53	*V* = 0.31
Poor sleep quality	12 (70.6%)	46 (73.0%)		

Data are presented as mean (standard deviation) or frequency (percentage). ^a^ Quantitative variables were compared using Student’s *t*-test for independent samples (migraine without TMD group vs. migraine with TMD group); qualitative variables were contrasted using X^2^ test. Bold *p*-values indicate statistically significant differences (*p* < 0.05). ^b^ Post hoc sensitivity analysis was performed using G*Power 3.1.9.6. The available sample size provided sufficient power to detect only large effects for Student’s *t* tests and moderate-to-large effects for χ^2^ tests, based on Cohen’s d (*d*) and Cramér’s V (*V*), respectively. TMD: temporomandibular disorders; STAI-S: State–Trait Anxiety Inventory—state subscale; STAI-T: State–Trait Anxiety Inventory (STAI)—trait subscale; S-DEP: State-Trait Depression Questionnaire—state subscale; T-DEP: State-Trait Depression Questionnaire—trait subscale; PSQI: Pittsburgh Sleep Quality Index.

## Data Availability

The data that support the findings of this study are available on request from the corresponding author. The data are not publicly available due to privacy or ethical restrictions.

## Supplementary Materials

The following supporting information can be downloaded at: https://www.mdpi.com/article/10.3390/jcm15155889/s1.

## Abbreviations

The following abbreviations are used in this manuscript:

95% CI	95% confidence interval
ANOVA	One-way analysis of variance
CM	Chronic migraine
DALYs	Disability-adjusted life years
DC/TMD	Diagnostic Criteria for Temporomandibular Disorders
EM	Episodic migraine
HIT-6	Headache Impact Test
ICHD-beta	Beta version of the third International Classification of Headache Disorders
PR	Prevalence ratio
PSQI	Pittsburgh Sleep Quality Index
SD	Standard deviations
STAI	State-Trait Anxiety Inventory,
ST-DEP	State-Trait Depression Questionnaire
TMD	Temporomandibular Disorders
