# Supplementary material for: Comorbidity of Temporomandibular Disorders, Anxiety, Depression, and Poor Sleep Quality in Episodic and Chronic Migraine: A Cross-Sectional Observational Study
Source: J Clin Med. 2026 Jul 28;15(15):5889. doi: 10.3390/jcm15155889 (PMC13466288; doi:10.3390/jcm15155889)
Supplement: Supplementary file 1 [file jcm-15-05889-s001.zip › jcm-4420182-supplementary.pdf]

Supplementary files. Logistic regression secondary analysis to adjust to headache frequency. The analysis of psychosocial variables association with the subgroups of migraine with or without TMD.

	B (SE)	P value	Odds Ratio	95%CI for Odds ratio	
				Lower	Upper
**HIT-6**					
Little or no impact, Some impact or Considerable impact[a]		ref.			
Severe impact	-0.07 (0.62)	0.92	0.94	0.28	3.13
**Headache days/month**	-0.11 (0.04)	0.008	0.90	0.83	0.97
**Constant**	-0.13 (0.53)	0.81	0.88		

R^2 = 0.05 (Hosmer &Lemeshow), 0.12 (Cox&Snell), 0.19 (Nagelkerke). Model $X^2(2)$ = *10.445, p=.005*.

[a]categories were grouped due to zero observation in more than one cell.

	B (SE)	P value	Odds Ratio	95%CI for Odds ratio	
				Lower	Upper
**STAI-S**		0.93			
Mild			ref.		
Low-moderate	-0.53 (0.93)	0.57	0.59	0.10	3.62
Moderate-high	-0.34 (0.83)	0.68	0.71	0.14	3.61
High	-0.04 (0.72)	0.95	0.96	0.23	3.91
**Headache days/month**	-0.11 (0.046)	0.007	0.90	0.83	0.97
**Constant**	-0.01 (0.55)	0.98			

R^2 = 0.19 (Hosmer &Lemeshow), 0.13 (Cox&Snell), 0.20 (Nagelkerke).

Model $X^2(4)$= *10.896, p=0.03.*

	B (SE)	P value	OR	95%CI for Odds ratio	
				Lower	Upper
**STAI-T**		0.26			
Mild			ref.		
Low-moderate	-1.16 (0.79)	0.14	0.31	0.07	1.49
Moderate-high	-0.92 (0.80)	0.26	0.40	0.08	1.99
High	-1.48 (0.90)	0.10	0.23	0.04	1.32
**Headache days/month**	-0.11 (0.04)	0.01	0.90	0.83	0.97
**Constant**	0.54 (0.57)	0.35	1.71		

R^2 = 0.12 (Hosmer &Lemeshow), 0.16 (Cox&Snell), 0.26 (Nagelkerke).

Model $X^2(4)$= *14.551, p=0.006*

	B (SE)	P value	Odds Ratio	95%CI for Odds ratio Lower	Upper
**S-DEP**		0.83			
Mild			ref.		
Low-moderate	0.05 (0.76)	0.95	1.05	0.24	4.64
Moderate-high	-0.60 (0.99)	0.54	0.55	0.08	3.79
High	-0.50 (0.81)	0.44	0.61	0.13	2.96
**Headache days/month**	-0.11 (0.04)	0.008	0.90	0.83	0.97
**Constant**	-0.03 (0.64)	0.96	1.03		

R^2 = 0.28(Hosmer &Lemeshow), 0.13 (Cox&Snell), 0.21 (Nagelkerke).
Model *$X^2(4)$= 11.335, p=0.02*

	B (SE)	P value	Odds Ratio	95%CI for Odds ratio	
				Lower	Upper
**T-DEP**		0.79			
Mild			ref.		
Low-moderate	0.16 (0.73)	0.83	1.17	0.28	4.86
Moderate-high + High[a]	-0.34 (0.69)	0.62	0.71	0.18	2.75
**Headache days/month**	-0.11 (0.04)	0.008	0.90	0.83	0.97
**Constant**	-0.11 (0.56)	0.85	0.90		

R^2 = 0.25 (Hosmer &Lemeshow), 0.13 (Cox&Snell), 0.20 (Nagelkerke).
Model *$X^2(3)$= 10.909, p= 0.012.*
[a]categories were grouped due to zero observation in one cell.

	B (SE)	P value	Odds Ratio	95%CI for Odds ratio	
				Lower	Upper
**PSIQ classification**					
Good sleep quality			ref.		
Poor sleep quality	0.20 (0.64)	0.76	1.22	0.34	4.29
**Headache days/month**	-0.11 (0.04)	0.006	0.89	0.82	0.97
**Constant**	-0.28 (0.59)	0.64	0.76		

R^2 = 0.08 (Hosmer &Lemeshow), 0.12 (Cox&Snell), 0.19 (Nagelkerke).
Model *$X^2(2)$= 10.527, p=0.005*
